# Resveratrol Modulates Cytokine-Induced JAK/STAT Activation More Efficiently than 5-Aminosalicylic Acid: An *In Vitro* Approach

**DOI:** 10.1371/journal.pone.0109048

**Published:** 2014-10-01

**Authors:** Diana Serra, Ana T. Rufino, Alexandrina F. Mendes, Leonor M. Almeida, Teresa C. P. Dinis

**Affiliations:** 1 CNC – Center for Neuroscience and Cell Biology, University of Coimbra, Coimbra, Portugal; 2 Faculty of Pharmacy, University of Coimbra, Coimbra, Portugal; National Cancer Institute, United States of America

## Abstract

**Background:**

Many advances have been recently made focused on the valuable help of dietary polyphenols in chronic inflammatory diseases. On the other hand, current treatment options for intestinal bowel disease patients are unsatisfying and, for this reason, it is estimated that many patients use dietary supplements to achieve extra benefits.

**Aim:**

The aim of this work was to analyze under a mechanistic perspective the anti-inflammatory potential of resveratrol, a natural polyphenolic compound, and to compare it with a pharmaceutical agent, 5-aminosalicylic acid, using the intestinal HT-29 cell line, as a cellular model.

**Methodology and Principal Findings:**

In the present study, HT-29 colon epithelial cells were pre-treated with 25 µM resveratrol and/or 500 µM 5-aminosalicylic acid and then exposed to a combination of cytokines (IL-1α, TNF-α, IFN-γ) for a certain period of time. Our data showed that resveratrol, used in a concentration 20 times lower than 5-aminosalicylic acid, was able to significantly reduce NO and PGE_2_ production, iNOS and COX-2 expression and reactive oxidant species formation induced by the cytokine challenge. However, as already verified with 5-aminosalicylic acid, in spite of not exhibiting any effect on IkB-α degradation, resveratrol down-regulated JAK-STAT pathway, decreasing the levels of activated STAT1 in the nucleus. Additionally, resveratrol decreased the cytokine-stimulated activation of SAPK/JNK pathway but did not counteract the cytokine-triggered negative feedback mechanism of STAT1, through p38 MAPK.

**Conclusion/Significance:**

Taken together, our results show that resveratrol may be considered a future nutraceutical approach, promoting remission periods, limiting the inflammatory process and preventing colorectal cancer, which is common in these patients.

## Introduction

In the last decades, many studies have shed light on the impact of dietary polyphenols in chronic inflammatory diseases, namely diabetes [1]–[3], atherosclerosis [2], [4] and inflammatory bowel diseases [5], [6]. Although the cell signaling mechanisms involved are far from being fully understood, many recent studies believe that the consumption of these biological phytochemicals can be truly advantageous to avoid or limit diseases progression [2], [3], [7]–[10]. For instance, some epidemiological studies show that a moderated intake of red wine (rich in polyphenols) can be useful in the prevention of cardiovascular diseases [11].

**Figure 1:**
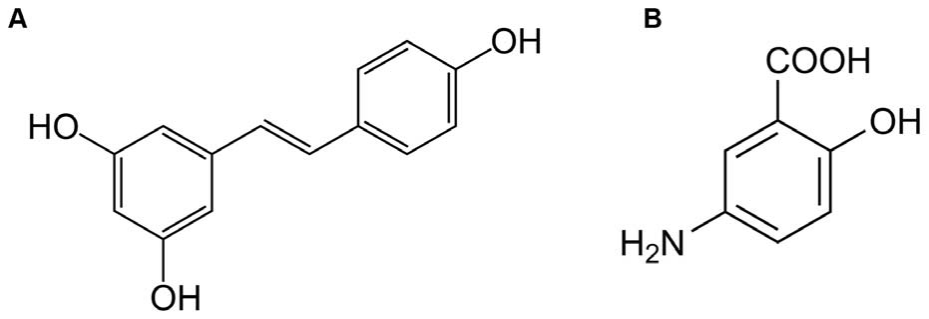
Chemical structures of resveratrol (A) and 5-aminosalicylic acid (B).

Resveratrol (3,5,4′-trihydroxy-trans-stilbene) is a natural non-flavonoid polyphenol, found mainly in grapes and red-wine and is one of the most studied polyphenols [10], [12]–[15]. However, the major concerns about resveratrol (Resv) efficacy are related to its low oral bioavailability [16], [17] and to the possible induction of liver damage [18]. Nevertheless, previous reports demonstrated that resveratrol has an important role as an anti-inflammatory agent [6], [14], [19], [20] and considering that the intestine is a target site for resveratrol action, it is of great interest to further understand the beneficial effects of resveratrol in an intestinal disease which is mainly characterized by inflammation, as the inflammatory bowel disease (IBD). IBD is a chronic inflammatory disorder of the gastrointestinal tract, which includes Crohn's Disease and Ulcerative Colitis, characterized by periods of remission and of relapses, whose etiology remains enigmatic [21], [22]. Until now, this disease does not have cure and consequently the pharmacological treatment is used to prevent and treat symptoms and still to induce or maintain the remission periods. There has been a huge advance in the therapy options for IBD patients but the conventional therapies, as the well-known anti-inflammatory 5-aminosalicylic acid (5-ASA), remain the cornerstone of treatment for the majority of these patients [23]. Besides, since existing treatment options for IBD patients often bring marginal results, dietary supplements have deserved increasing interest to achieve extra benefits. Therefore, the advantages and implications of such dietary supplements for IBD patients need to be more elucidated. A previous work performed in our laboratory has focused on the anti-inflammatory potential of the flavonoid polyphenol, cyanidin-3-glucoside, in comparison with the active principle, 5-ASA, in the context of IBD [8]. The aim of the present study was to extend this research to a polyphenol with a completely different chemical structure, the resveratrol, and to explore, under a mechanistic perspective, its anti-inflammatory potential as compared to 5-ASA (Figure 1). For this purpose, the HT-29 cell line was used as a colon epithelial cells model, stimulated by a mixture of cytokines (Cyt). Cytokines are molecules rapidly released by injured tissues and are inducers of inflammatory response [24], [25]. Some previous studies have suggested that exposure of intestinal cells to a mixture of cytokines can activate inflammatory cascades (namely, NF-kB, MAPKs and JAK-STAT pathways) and in turn increase the expression of pro-inflammatory enzymes, (iNOS and COX-2), the production of pro-inflammatory mediators (NO and PGE_2_) and the formation of reactive oxidant species (ROS) [26]–[28]. The down-regulation of these pro-inflammatory cascades emerges as a valuable strategy in IBD, since they are usually heightened in these patients [29]–[32].

Our data demonstrated, for the first time, the stronger anti-inflammatory efficiency of Resv as compared to 5-ASA, given that 25 µM Resv was more effective than 500 µM 5-ASA in down-regulating the production of pro-inflammatory mediators (NO, PGE_2_), pro-inflammatory enzymes (iNOS, COX-2 mRNAs and proteins) and ROS formation induced by the cytokines. Moreover, as it was previously verified by some of us for 5-ASA [8], Resv did not affect IkB-α degradation, but significantly decreased the amount of activated STAT1 in the nucleus of cytokine-stimulated HT-29 cells. Besides, Resv, alone or in combination with 5-ASA, down-regulated STAT1 activation through a dependent p38 MAPK mechanism and inhibited the cytokine-induced SAPK/JNK activation. Therefore, our work gives a step forward unravelling JAK-STAT as well as MAPK signaling as key cascades involved in Resv anti-inflammatory protection and in its potential anticancer action.

## Materials and Methods

### Reagents

Resveratrol purified from natural sources was obtained from Extrasynthése (Genay, France). Its purity was above 95%, as measured by HPLC, and it was used as a solution in DMSO (5 mM) and stored at −20°C.

Laboratory chemicals namely dimethylsulfoxide (DMSO), sodium dodecyl sulfate (SDS), 2,3-diaminonaphthalene (DAN), 3-(4,5-dimethylthiazol-2yl)2,5-diphenyl-tetrazolium bromide (MTT), 2′,7′-dichlorodihydrofluorescein diacetate (DCFH-DA) phenylmethylsulfonyl fluoride (PMSF) Hoechst 33258, streptomycin/penicillin, protease inhibitor cocktail and phosphatase inhibitors were purchased from Sigma-Aldrich Co.

For cell culture, Dulbecco's modified Eagle's medium (DMEM), 0.25% trypsin, fetal bovine serum (FBS) and phosphate-buffered saline (PBS) pH 7.4, were obtained from Gibco-Invitrogen.

Rabbit polyclonal antibody to iNOS and goat polyclonal antibody to phospho-STAT1 (Tyr701) were purchased from Santa Cruz Biotechnology (Santa Cruz, CA, USA); rabbit monoclonal antibody to phospho-p38 MAPK (Thr180/Tyr182) and rabbit polyclonal antibodies to phospho-SAPK/JNK (Thr183/Thr185) and to IκB-α were purchased from Cell Signaling Technology (MA, USA); mouse monoclonal antibodies to β-actin and to β-tubulin were purchased from Sigma-Aldrich Co and rabbit polyclonal antibodies to COX-2, to lamin B1 and anti-rabbit, anti-mouse, anti-goat IgG secondary antibodies were obtained from Abcam (Cambridge, UK). The Alexa Fluor 594 chicken anti-goat IgG fluorescent secondary antibody was bought from Alfagene (Life Technologies).

IL-1α, TNF-α and IFN-γ were purchased from Invitrogen (NY, USA).

### Cell culture

Human colon cancer cell line (HT-29) and human liver carcinoma cell line (HepG2) were obtained from European Collection of Cell Cultures (Porton Down, Salisbury, UK). Both cell lines were grown in DMEM supplemented with 10% FBS, 100 U/ml penicillin and 100 µg/ml streptomycin at 37°C in a humidified atmosphere of 5% CO_2_. Cells were passaged weekly and sub-cultured at confluence. Before each experiment, cells at 80% confluence were starved in serum-free medium for 24 hours. Growth-arrested cultures, in medium without FBS, were treated according to the various experimental purposes.

HT-29 cells were stimulated with a combination of cytokines consisting of 10 ng/ml IL-1α, 20 ng/ml TNF-α and 60 ng/ml IFN-γ. Each cytokine was previously diluted in 1% BSA in PBS. HT-29 cells were pre-treated with Resv, 5-ASA or both for 1 hour before the exposure to the cytokines and then maintained with the inflammatory stimulus for different time periods, depending on the experiment.

### Cell Viability

Cell viability was assessed by the mitochondrial-dependent reduction of 3-(4,5-dimethylthiazol-2yl) 2,5-diphenyltetrazolium bromide (MTT) to formazan, which is directly proportional to the number of living cells. Cells in 6-well plates (8×10^5^ cells/well) were pre-treated with several concentrations of Resv, 500 µM 5-ASA or 25 µM Resv plus 500 µM 5-ASA for 24 hours. At the end, cells were washed with PBS and incubated with MTT (0.5 mg/ml) for 1 hour, at 37°C. Then, the medium was removed and the formazan crystals were dissolved in DMSO (900 µl). The extent of formazan formation was recorded at 530 nm in a Synergy HT plate reader.

Results were expressed as a percentage of control cells, *i.e.* non-treated cells.

### Measurement of Nitric Oxide Production

Nitric oxide production, in intestinal cells, was determined by measuring the amount of nitrite accumulated in cell culture supernatants. Nitrite was measured using a sensitive fluorimetric assay based upon the reaction of nitrite with 2,3-diaminonaphthalene (DAN), under acidic conditions, to form the fluorescent product 1-(H)-naphthotriazole [33]. Briefly, at the end of 24 hours of incubation, the supernatants were collected and nitrite was evaluated by adding 200 µl of freshly prepared DAN (0.025 mg/ml in 0.62 M HCl) to 200 µl of supernatant and mixed immediately. After 10 minutes of incubation at room temperature in the dark, the reaction was stopped with 100 µl of 3 M NaOH. A standard curve was produced with known concentrations of sodium nitrite. Fluorescence intensity was read in a dual wavelength spectrophotofluorimeter, with excitation and emission at 365 nm and 405 nm, respectively. The sensitivity of the assay is 10 nM.

### Assessment of Prostaglandin E_2_ Production

Confluent HT-29 cells grown on six-well plates (8×10^5^ cells/well) were treated as described in cell culture. After 16 hours of incubation time, supernatants were collected and processed for PGE_2_ quantification, by using a competitive immunoassay kit (PGE_2_ EIA Kit) from Enzo Life Science, according to the manufacturer's instructions. The values were related to protein content, as measured by the Bradford assay (Bio-Rad, USA).

### Western-blot Analysis

As previously described [8], total, cytoplasmic and nuclear cellular protein extracts from HT-29 cells were prepared and analyzed by Western-blotting. For total cellular protein extracts, washed cell pellets were resuspended in an ice-cold lysis buffer (50 mM Hepes pH 7.4, 150 mM NaCl, 2 mM EDTA, 10% (w/v) glycerol, 0.5% (w/v) sodium deoxycholate, 1% (v/v) Triton X-100, 1 mM PMSF, 1/100 (v/v) protease inhibitor cocktail) for 20 minutes, on ice. Cell debris was subsequently removed by centrifugation at 14000 rpm for 20 minutes at 4°C and supernatants were then collected and stored at −20°C. Cytoplasmic protein extracts were obtained essentially in the same way. Washed cells were lysed in an ice-cold buffer containing 10 mM Tris–HCl, 10 mM NaCl, 3 mM MgCl_2_, 0.5% Nonidet P-40 and 1% protease inhibitor cocktail, pH 7.5, for 5 minutes on ice. Afterwards, lysates were centrifuged at 5000 rpm for 5 minutes at 4°C and the supernatants (cytoplasmic extracts) were collected and stored at −20°C. The pellets were also collected and resuspended in an ice-cold buffer with 20 mM Hepes, 5 mM MgCl_2_, 0.2 mM EDTA, 1 mM DTT, 300 mM NaCl, 20% (w/v) glycerol, and 1% protease inhibitor cocktail, pH 7.5 and left on ice for 30 minutes. Then, the mixture was centrifuged at 14000 rpm for 20 minutes at 4°C and the supernatants (nuclear extracts) were saved at −80°C.

Protein concentration was determined by using the Bio-Rad protein assay reagent (Bradford assay), according to the manufacturer's specifications (Bio-Rad, USA).

A range of 30–80 micrograms of reduced and denatured proteins were separated by SDS/PAGE electrophoresis on a 10%–12% (v/v) acrylamide gel and transferred onto polyvinylidene difluoride (PVDF) membranes (Amersham Biosciences, UK) by electroblotting. To avoid non-specific binding, membranes were blocked with skimmed milk in TBS pH 7.6 supplemented with 0.1% (v/v) Tween 20 (TBS-T: 20 mM Tris–HCl, 150 mM NaCl, 0.1% Tween 20) and then probed with antibodies against iNOS, COX-2 and IκB-α, overnight at 4°C and against phospho-STAT1, phospho-p38 MAPK and phospho-SAPK/JNK 3 hours at room temperature, with a constant low shaking. After finishing, membranes were washed three times and further incubated with alkaline phosphatase-conjugated secondary antibodies (2 hours at room temperature and constant shaking). Immunoreactive bands were detected by fluorescence in a Typhoon 9000 scanner (Amersham Biosciences) and analyzed with the ImageQuant software from Amersham Biosciences. After analysis of target proteins, each blot was stripped off and reprobed with the primary antibodies against β-actin, β-tubulin or lamin B1, used as controls for protein loading.

### Total RNA extraction and quantitative real-time RT-PCR (qRT-PCR)

Total RNA was extracted from HT-29 cells seeded in six-well-plates (8×10^5^ cells/well), after 1 hour of pretreatment with Resv and/or 5-ASA followed by 6 hours of cytokine-challenge, using the RNA extraction kit Aurum Total RNA Mini (Bio-Rad, Hercules, CA, USA), according to the manufacturer's instructions. Extracted RNA was quantified using a NanoDrop ND-1000 spectrophotometer at 260 nm and its purity and integrity were assessed by ratio of absorbance at 240/260 and 280/260 nanometers. A constant amount of RNA (1 µg/sample) was reverse transcribed into cDNA, using the NZY First-Stand cDNA Synthesis Kit (NZYtech, Portugal), according to the manufacturer's protocol. PCR reactions were performed with 25 µg/ml of transcribed cDNA. The primers for iNOS, COX-2 and the housekeeping gene HPRT-1 (hypoxanthine phosphoribosyltransferase-1) were designed using the Beacon Designer software (PREMIER Biosoft International, Palo Alto, CA) and the primers sequences were: iNOS, sense 5′- AATCCAGATAAGTGACATAAG -3′, antisense 5′- CTCCACATTGTTGTTGAT -3′; COX-2, sense 5′- ATTATGAGTTTATGTGTTGAC -3′; antisense 5′- TAGGAGAGGTTAGAGAAG -3′; HPRT-1 sense 5′- TGACACTGGCAAAACAATG -3′, antisense 5′- GGCTTATATCCAACACTTCG -3′. Real time-PCR was performed in 20 µl of total volume, containing 2 µl of each primer (250 nM), 2 µl of cDNA of each sample, 10 µl of the IQ SYBR Green Supermix (Bio-Rad) and RNase-free distilled water to make up the volume to 20 µl, in a CFX96 Real-Time PCR Detection System (Bio-Rad, Hercules, CA, USA). Thermal cycling conditions were the following: 3 minutes at 95°C to activate the iTaq DNA polymerase, then 45 cycles, each consisting of a denaturation step (95°C, 10 seconds), an annealing step (55°C, 30 seconds) and an elongation step (72°C, 30 seconds). Fluorescence measures were taken every cycle at the end of the annealing step and the specificity of the amplification products was evaluated through the analysis of the melting curve. The efficiency of the amplification reaction for each gene was calculated by running a standard curve of serially diluted cDNA sample. Gene expression was analyzed using the Bio-Rad CFX Manager 3.0 software (Bio-Rad, Hercules, CA, USA), which enables the analysis of the results with the Pfaffl method. The results for each gene of interest were normalized against HPRT-1, the housekeeping gene found to be stable under experimental conditions and expressed as a percentage of control cells, *i.e.* cytokine-stimulated cells.

### Fluorescence Confocal Microscopy

HT-29 colon epithelial cells were seeded onto glass coverslips on 24 well plates (1.5×10^5^ cells/well) and treated with Resv and/or 5-ASA for 1 hour and then exposed to the cocktail of cytokines for 30 minutes. After this period, the cells were washed with PBS and fixed with 4% paraformaldehyde in PBS for 20 minutes at room temperature. Fixed cells were washed with PBS and permeabilized with 0.2% Triton X-100 in PBS for 2 minutes. After washing, cells were incubated overnight with phospho-STAT1 antibody diluted in PBS (1∶50) at 4°C. Then, the cells were washed twice with PBS, followed by incubation with Alexa Flour 594 conjugated secondary antibody diluted in PBS (1∶100) for 4 hours at room temperature. After washing twice with PBS, the coverslips were mounted with glycerol and PBS containing the nucleic acid stain Hoechst (1 µg/ml). Cells were examined under a confocal microscope (Ziess LSM 510Meta).

### Evaluation of Intracellular Reactive Species

Intracellular reactive species were assessed by using the non-fluorescent probe 2′,7′-dichlorodihydrofluorescein diacetate (DCFH_2_-DA), which permeates cell membranes and may be oxidized by reactive species, yielding the fluorescent 2′,7′-dichlorofluorescein (DCF) [34]. Briefly, cells in 12-well plates (4×10^5^ cells/well) were previously incubated in the presence or in the absence of Resv and 5-ASA and further subjected to the combination of cytokines for 16 hours. After that period of time the cells were incubated with 5 µM DCFH_2_-DA in DMSO, at 37°C, in the dark for 15 minutes. Cells were then washed with PBS and maintained in 0.5 ml of PBS during the fluorescence intensity measurements in a Synergy HT plate reader (Bio-Tek Instruments) (excitation and emission wavelengths at 485 and 530 nm, respectively). Cells were also observed in an inverted fluorescence microscope (Zeiss Axiovert 40), using a FITC filter.

### Statistical Analysis

All data were expressed as means ± SEM of at least 3 independent assays, each one in duplicate. Differences between groups were analyzed by one-way analysis of variance (ANOVA) and Tukey's *post hoc* test was used as appropriate. Values of *p*<0.05 were accepted as statistically significant.

## Results

### 1. Resveratrol up to 25 µM did not affect HT-29 cells viability

**Figure 2:**
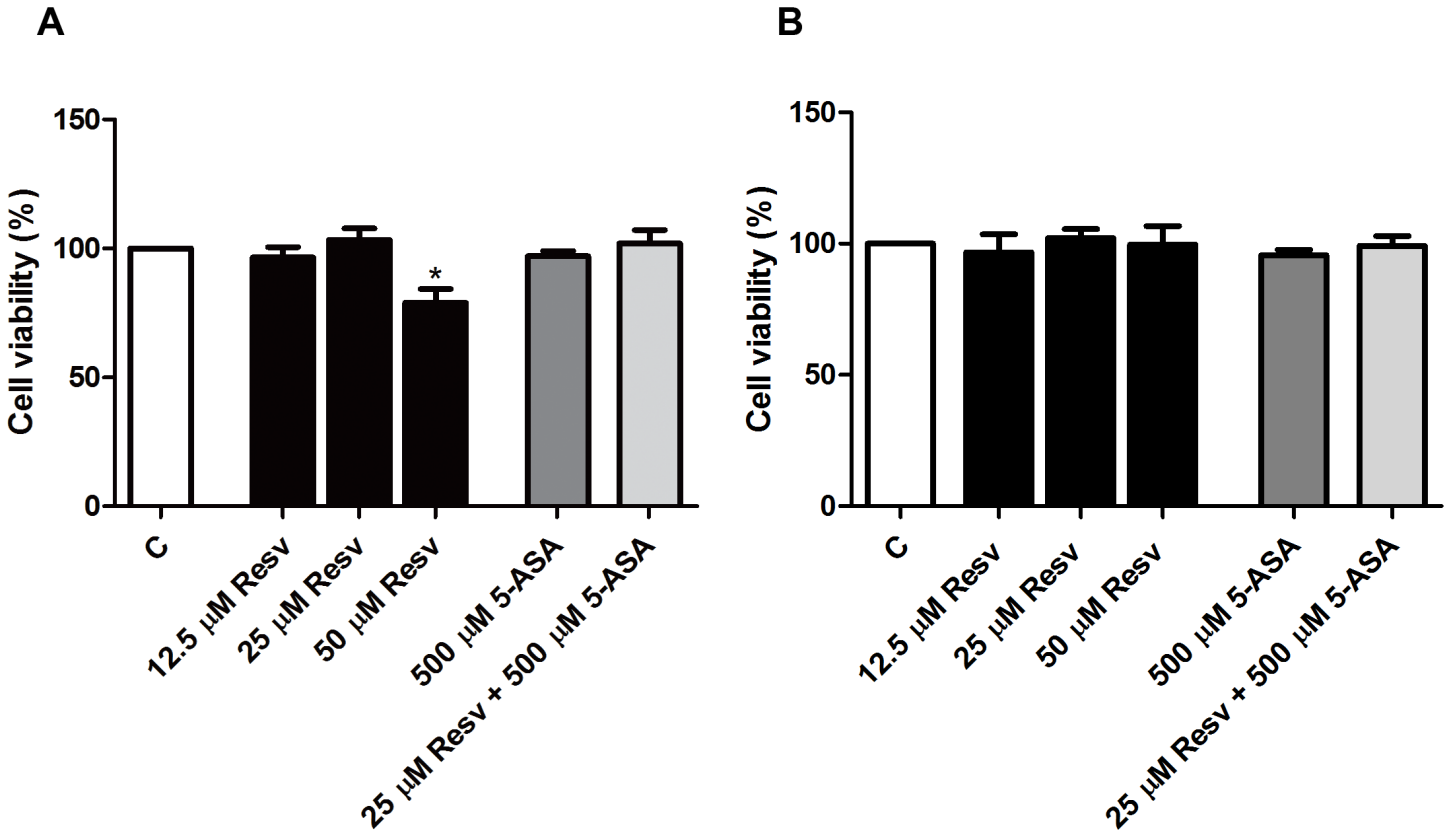
Effects of resveratrol and/or 5-ASA on cell viability. Cells were incubated for 24 hours with different concentrations of Resv (12.5 to 50 µM), 5-ASA (500 µM) and a combination of 25 µM Resv and 500 µM 5-ASA. (A) HT-29 cell-line viability and (B) HepG2 cell-line viability were assessed by MTT reduction and determined as percentage of control cells (without compounds). Values are mean ± SEM of at least three independent experiments, each one in duplicate. ^*^ *P*<0.05 vs Control.

The cytotoxicity of Resv alone or in combination with 5-ASA on HT-29 cell line was evaluated, upon 24 hours of cell incubation with the compounds, by the MTT assay. As illustrated in Figure 2A, neither Resv alone, in the concentration range of 12.5 to 25 µM, nor the combination of 25 µM Resv with 500 µM 5-ASA exerted cytotoxicity in HT-29 cells. However, at the concentration of 50 µM, Resv produced a small decrease in the cell viability relative to the control (untreated cells). Thus, concentrations of 25 µM Resv and 500 µM 5-ASA were chosen to perform the next experiments, since they proved to be subtoxic concentrations. As previously reported [8], the mixture of cytokines, at the concentrations selected as inflammatory stimulus in HT-29 cells, induced a decrease in cell viability to about 50 per cent (data not shown).

Considering that Resv in high doses may be hepatotoxic, we assessed the toxicity of Resv, 5-ASA and of both compounds in HepG2 cells as an in vitro model of human hepatocytes, in the same assay conditions. As illustrated in Figure 2B, none of the compounds induced loss of cell viability during this period of time.

### 2. Secretion of NO and PGE_2_ was inhibited more efficiently by Resveratrol than by 5-ASA, in stimulated HT-29 cells

To elucidate the ability of Resv alone or in combination with 5-ASA to inhibit the production of some pro-inflammatory mediators, the levels of NO and PGE_2_ generated by cytokine-stimulated HT-29 cells were evaluated.

**Figure 3:**
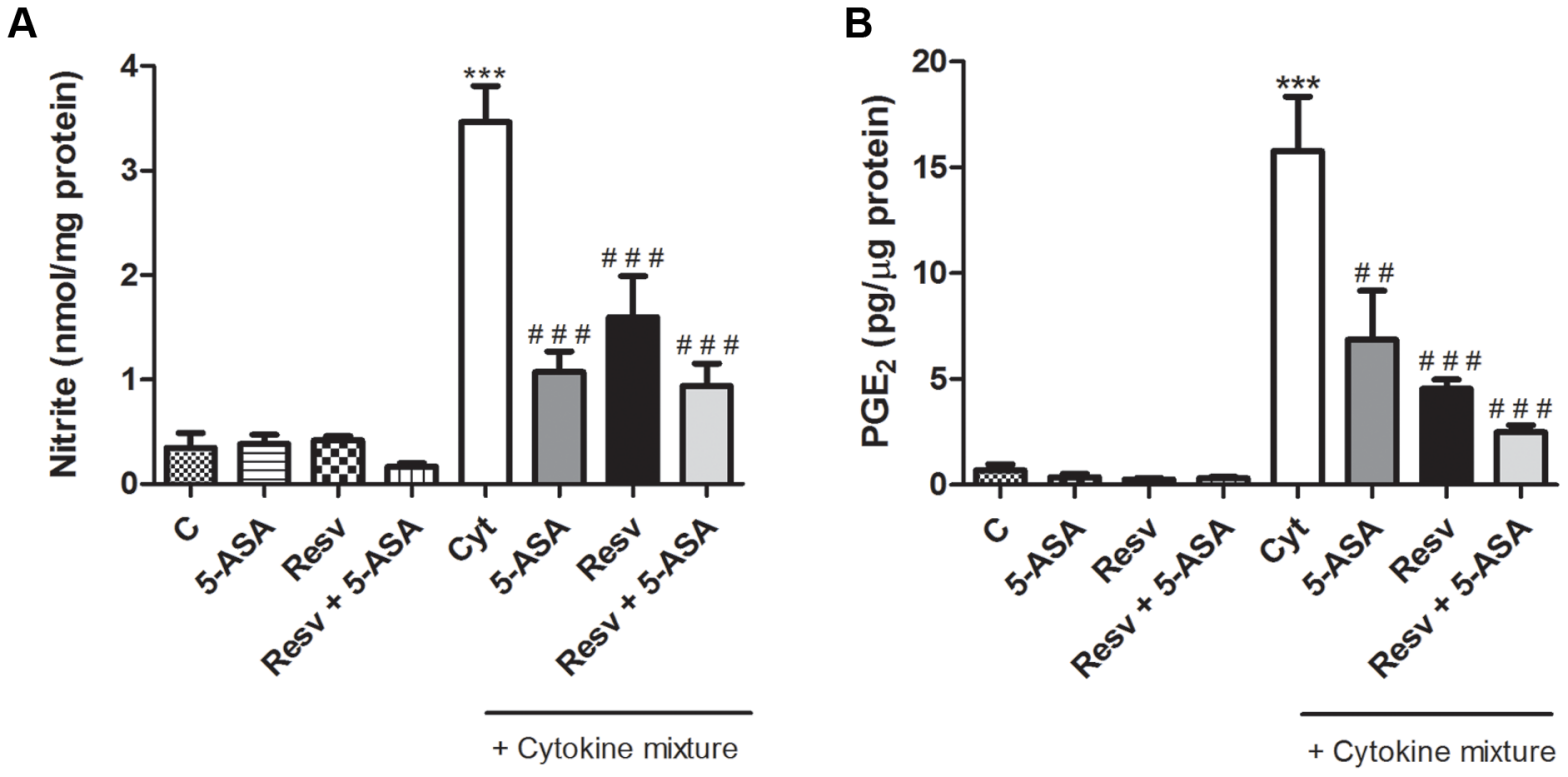
Resveratrol decreases cytokine–induced pro-inflammatory mediators production more efficiently than 5-ASA, in HT-29 cells. Cells were pre-incubated with 25 µM Resv or 500 µM 5-ASA or both (25 µM Resv plus 500 µM 5-ASA) and then exposed to cytokines (Cyt) for 24 hours or 16 hours for NO and PGE_2_, respectively. The NO (A) and PGE_2_ (B) production in cells was measured as described in “Materials and Methods”. Values are mean ± SEM of at least three independent experiments, each one in duplicate. ^***^ *P*<0.001 vs Control, ^##^ *P*<0.01, ^###^ *P*<0.001 vs Cytokines.

As shown in Figure 3A, stimulation of HT-29 cells with cytokines, for 24 hours, triggered a significant increase of cellular nitrite formation as compared to control (untreated cells), in agreement with previous studies. Cells treatment with 25 µM Resv, for 1 hour, before cytokine stimulation, significantly reduced the nitrite levels by about 50%. This inhibitory effect was not significantly different to that of 500 µM 5-ASA and occurred at a concentration 20 times lower. Also, the combination of 25 µM Resv with 500 µM 5-ASA did not cause any additional significant effect.

In order to draw comparison between Resv and 5-ASA on PGE_2_ production, cells were treated with the compounds for 1 hour and then exposed to the cytokine mixture (IL-1α, TNF-α and IFN-γ) for 16 hours. In Figure 3B, it is patent that PGE_2_ production was enhanced in response to cytokine treatment and that this increase was deeply inhibited by Resv by almost 75%, a higher inhibitory effect than that induced by 5-ASA (about 50%) at a concentration 20 times higher. The combined effect of Resv and 5-ASA seemed to be stronger than that of the individual compounds, however data are not statistically different.

### 3. Resveratrol counteracted, in a greater extent than 5-ASA, cytokine-stimulated expression of iNOS and COX-2 proteins and mRNAs, in HT-29 cells

**Figure 4:**
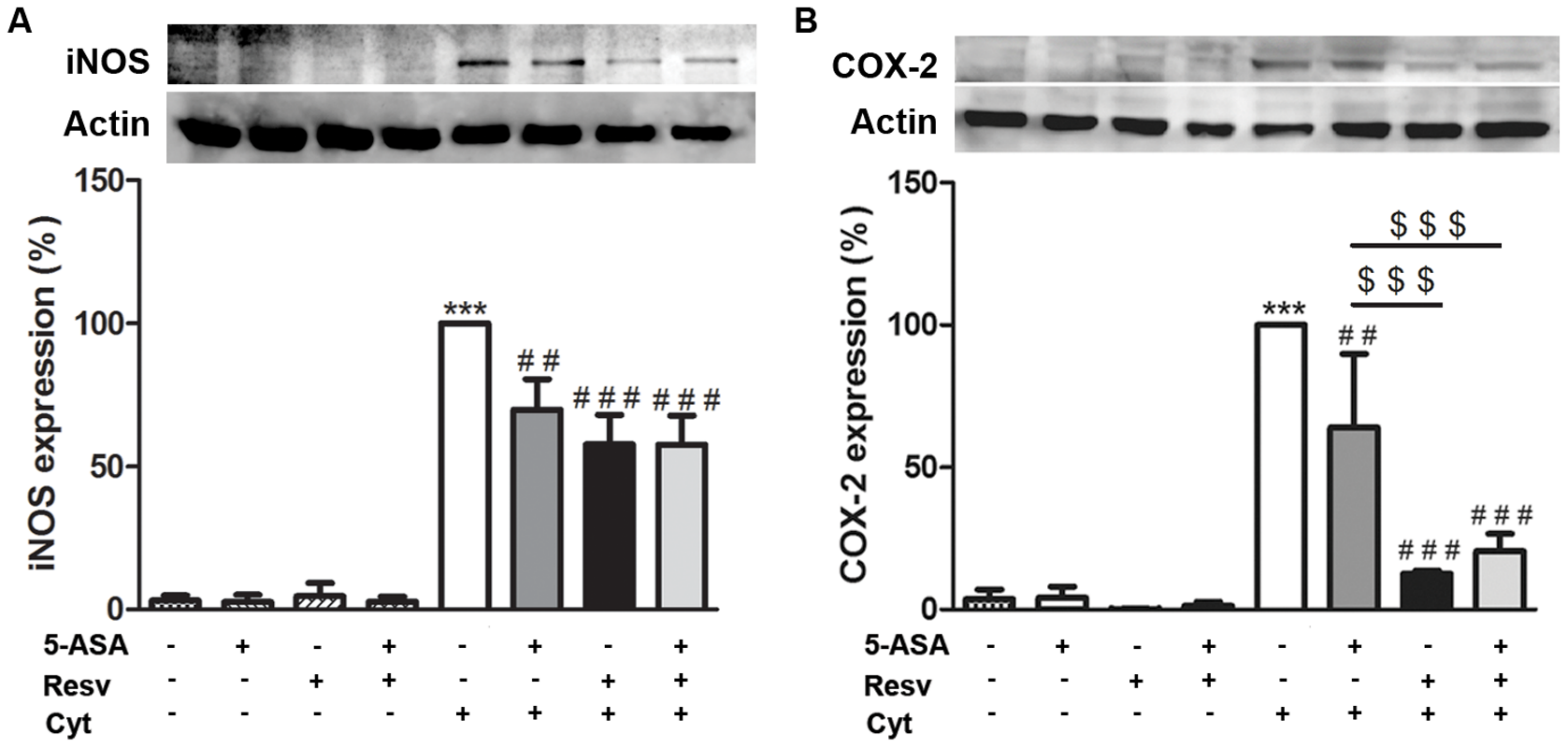
Resveratrol suppresses cytokine-induced iNOS and COX-2 expression more efficiently than 5-ASA, in HT-29 cells. Cells were pre-incubated with 25 µM Resv or 500 µM 5-ASA or both (25 µM Resv plus 500 µM 5-ASA) and then challenged with a combination of cytokines. iNOS (A) and COX-2 (B) expressions were evaluated after 24 hours or 16 hours, respectively, in total extracts by Western blotting, as described in “Materials and Methods”, and expressed as percentage of cytokine-stimulated cells. Values are mean ± SEM of at least three independent experiments, each one in duplicate. ^***^ *P*<0.001 vs Control, ^##^ *P*<0.01, ^###^ *P*<0.001 vs Cytokines and ^$$$^ *P*<0.001 vs ASA plus Cytokines.

**Figure 5:**
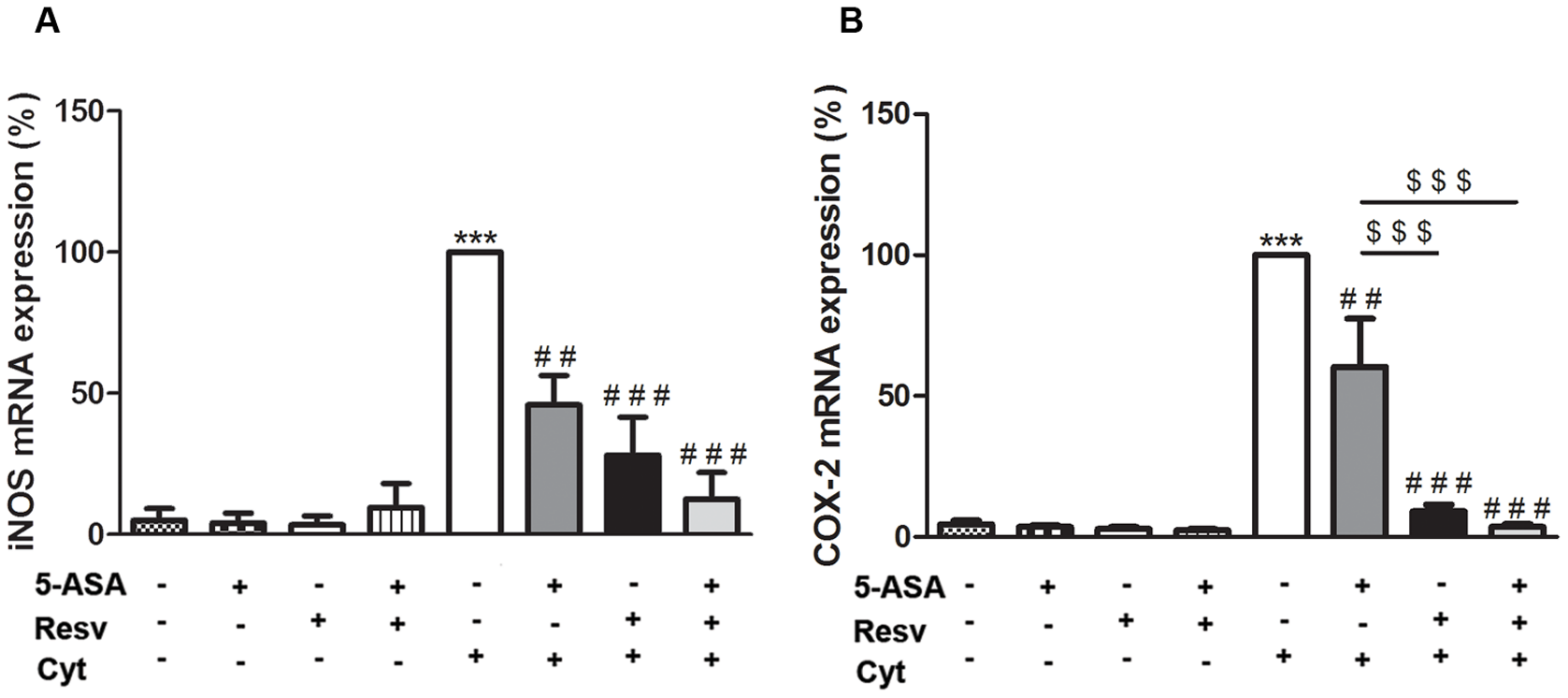
Resveratrol suppresses cytokine-induced iNOS and COX-2 mRNA levels more efficiently than 5-ASA, in HT-29 cells. Cells were pre-incubated with 25 µM Resv or 500 µM 5-ASA or both (25 µM Resv plus 500 µM 5-ASA) and then exposed to a combination of cytokines. iNOS (A) and COX-2 (B) mRNA production was evaluated after 6 hours by qRT-PCR, as described in “Materials and Methods”, and expressed as percentage of cytokine-stimulated cells. Values are mean ± SEM of at least three independent experiments, each one in duplicate. ^***^ *P*<0.001 vs Control, ^##^ *P*<0.01, ^###^ *P*<0.001 vs Cytokines and ^$$$^ *P*<0.001 vs ASA plus Cytokines.

To further investigate whether the protection afforded by Resv, with regarding to the pro-inflammatory mediators studied, was related to the inhibition of the inducible forms of NO synthase and of cyclooxygenase, the protein expressions and mRNA levels were determined by Western blotting and qRT-PCR, respectively. Analysing Figures 4 and 5, it is clear that protein and mRNA levels of iNOS and COX-2, which were hardly detectable in non-stimulated cells, were significantly enhanced after cytokine exposure, according to data previously reported [8]. However, cytokine stimulatory effect was significantly reduced by pre-treating the cells with Resv and/or 5-ASA, for 1 hour, before the exposure to cytokines. As illustrated in Figures 4 and 5, the extent to which Resv alone counteracted the cytokine-induced increase in COX-2 protein and mRNA levels was clearly and statistically higher than that assigned to 5-ASA, particularly if the different concentrations of the compounds are taken into account (25 µM Resv and 500 µM 5-ASA). However, the combination of Resv with 5-ASA did not promote an enhancement of this effect.

### 4. Resveratrol *per se* or in combination with 5-ASA did not prevent cytokine-induced IkB-α degradation, in HT-29 cells

**Figure 6:**
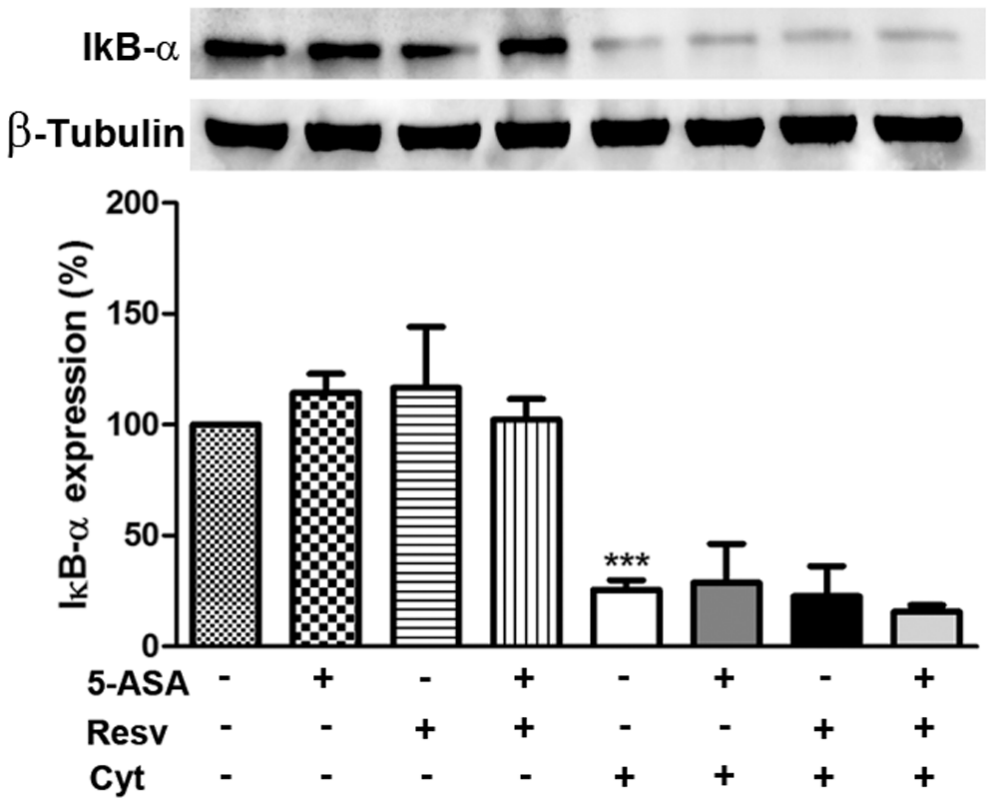
Resveratrol does not prevent IkB-α degradation induced by cytokines, in HT-29 cells. Cells were pre-incubated with 25 µM Resv or 500 µM 5-ASA or both (25 µM Resv plus 500 µM 5-ASA) and then exposed to a combination of cytokines for 30 minutes. IkB-α degradation was analyzed in cytoplasmic extracts by Western blotting, as described in “Materials and Methods”, and expressed as percentage of control cells, i.e. cells not treated. Values are mean ± SEM of at least three independent experiments, each one in duplicate. ^***^ *P*<0.001 vs Control.

In order to verify the involvement of NF-kB pathway in the protection afforded by Resv alone or in combination with 5-ASA, cells were stimulated with cytokines in the absence and presence of the compounds and the degradation of IkB-alpha was analyzed by Western blotting. As previously observed [8], 30 minutes after cells stimulation, the mixture of cytokines induced the degradation of IkB-α, resulting in a decrease of this protein to about 75% relative to a control (non-treated cells). As shown in Figure 6, Resv was not able to prevent the degradation of IkB-α, either alone or associated with 5-ASA.

### 5. Resveratrol inhibited expression of pro-inflammatory markers in cytokine-stimulated HT-29 cells via JAK-STAT pathway

**Figure 7:**
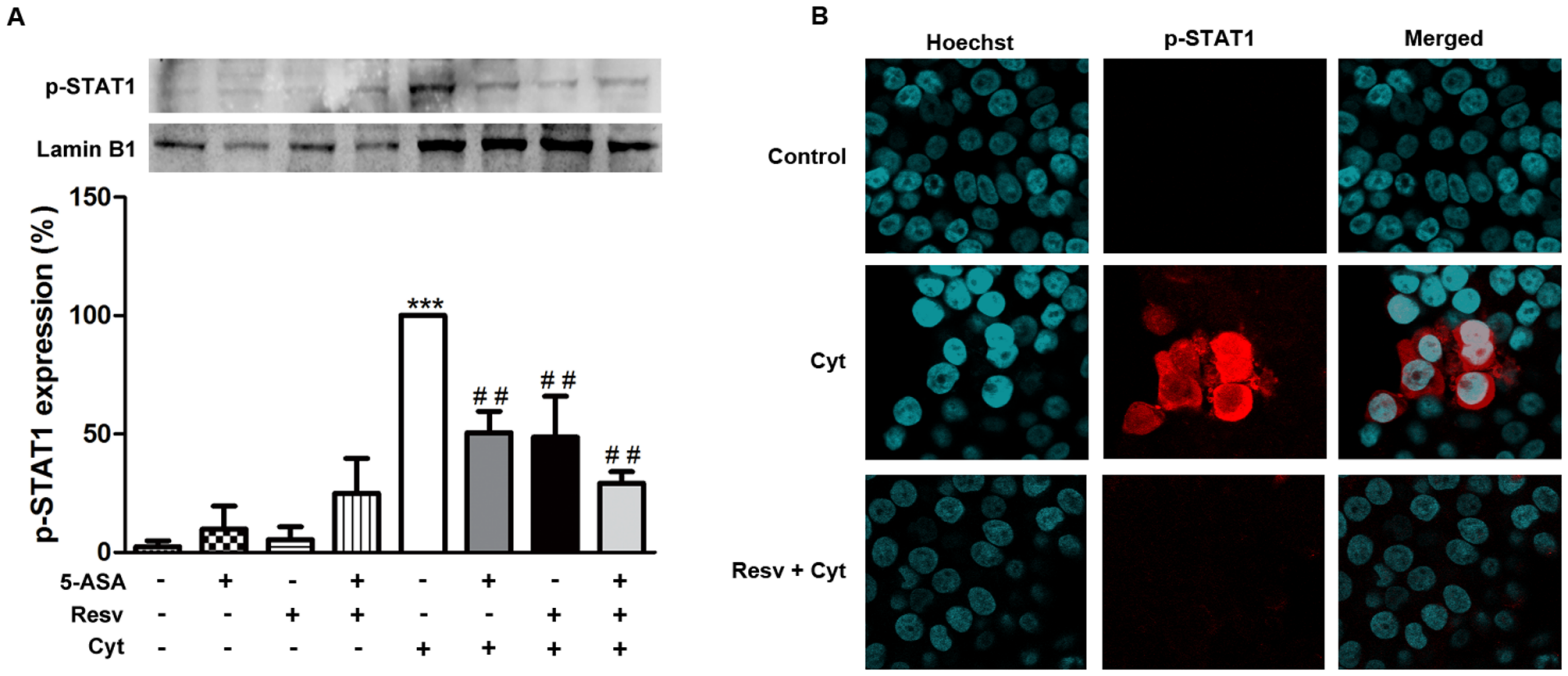
Resveratrol decreases activated-STAT1 levels in the nucleus of cytokine-stimulated HT-29 cells more efficiently than 5-ASA. Cells were pre-incubated with 25 µM Resv or 500 µM 5-ASA or both (25 µM Resv plus 500 µM 5-ASA) and then exposed to a combination of cytokines for 30 minutes. The levels of Tyr701 phospho-STAT1 were analyzed in nuclear extracts by Western blotting (A), as described in “Materials and Methods” and expressed as percentage of cytokine-stimulated cells. Values are mean ± SEM of at least three independent experiments, each one in duplicate. ^***^ *P*<0.001 vs Control and ^##^ *P*<0.01 vs Cytokines. (B) Representative confocal microscopy pictures of non-stimulated, cytokine-stimulated and Resv pre-incubated HT-29 cells. Simultaneous DNA labelling with Hoechst was performed to visualize the nuclear compartments.

In pursuit of knowing more about the mechanisms underlying the protection afforded by Resv against inflammation, in our experimental conditions, further pathways beyond NF-kB were explored, namely that involving the transcription factor STAT1. Thus, the ability of Resv to decrease the levels of the tyrosine (Tyr)701 phosphorylated form of this transcription factor in the nucleus was monitored. As verified in Figure 7A, Resv was able to inhibit the cytokine-induced levels of activated (Tyr701 phosphorylated) STAT1 in the nucleus in a similar way to 5-ASA, but at a concentration 20 times lower. The association of Resv with 5-ASA did not elicit an increase in efficiency in this pathway. In order to further illustrate the ability of the compounds under study in decreasing the amount of Tyr701 phospho-STAT1 in the nucleus of cytokine-stimulated HT-29 cells, immunocytochemical studies were performed. Representative confocal images of HT-29 cells shown in Figure 7B denoted nuclear staining patterns consistent with the results of immunoblotting of the nuclear extracts. Microscopic data obtained with 5-ASA plus cytokines and with the combination (Resv and 5-ASA) plus cytokines were very similar to those depicted in Figure 7B for Resv alone plus cytokines and, thus, they were not represented, for the sake of clarity.

### 6. The protection afforded by Resveratrol alone or in combination with 5-ASA against cytokine-induced inflammation involved MAPKs signaling, in HT-29 cells

**Figure 8:**
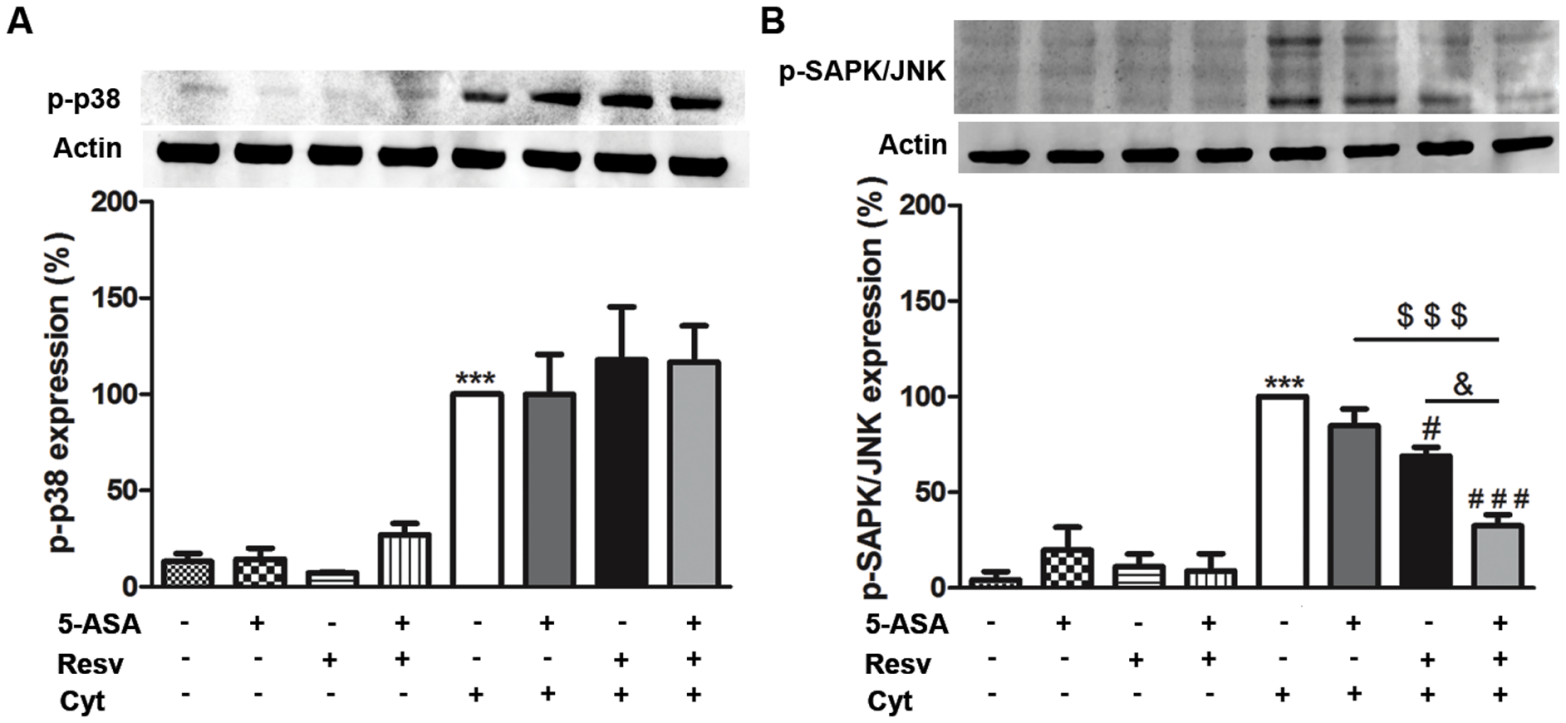
p38 MAPK and SAPK/JNK are involved in resveratrol protection, alone or plus 5-ASA, in cytokine-stimulated HT-29 cells. Cells were pre-incubated with 25 µM Resv or 500 µM 5-ASA or both (25 µM Resv plus 500 µM 5-ASA) and then exposed to a combination of cytokines for 30 minutes. Phospho-p38 MAPK (A) and phospho-SAPK/JNK (B) expressions were evaluated in total extracts by Western blotting, as described in “Materials and Methods”, and expressed as percentage of cytokine-stimulated cells. Values are mean ± SEM of at least three independent experiments, each one in duplicate. ^***^ *P*<0.001 vs Control (non-treated cells) ^#^ *P*<0.05, ^###^ *P*<0.001 vs Cytokines, ^$$$^ *P*<0.001 vs ASA plus Cytokines and ^&^ *P*<0.05 vs Resv plus Cytokines.

Being aware that the upstream kinases, such as p38 and SAPK/JNK MAPKs, play an important role in the regulation of the activation of several transcription factors, we also evaluated the effects of Resv, alone or in combination with 5-ASA, on p38 MAPK and SAPK/JNK phosphorylation (activation). It was observed (Figure 8A and 8B) that the combination of cytokines, after 30 min of cell-stimulation, induced the phosphorylation of p38 MAPK and of SAPK/JNK. Resv alone or in combination with 5-ASA maintained the levels of activated p38 MAPK in HT-29 cells (Figure 8A), but counteracted the cytokine-induced activation of SAPK/JNK, in those cells (Figure 8B). On the other hand, 5-ASA alone, in such activated cells, did not affect neither p38 MAPK nor SAPK/JNK activation. However, cells pre-incubation with 5-ASA plus Resv significantly increased the suppressive effect of Resv on SAPK/JNK phosphorylation.

### 7. Resveratrol, unlike 5-ASA, exerted a strong inhibition in the generation of intracellular reactive species, in cytokine-stimulated HT-29 cells

**Figure 9:**
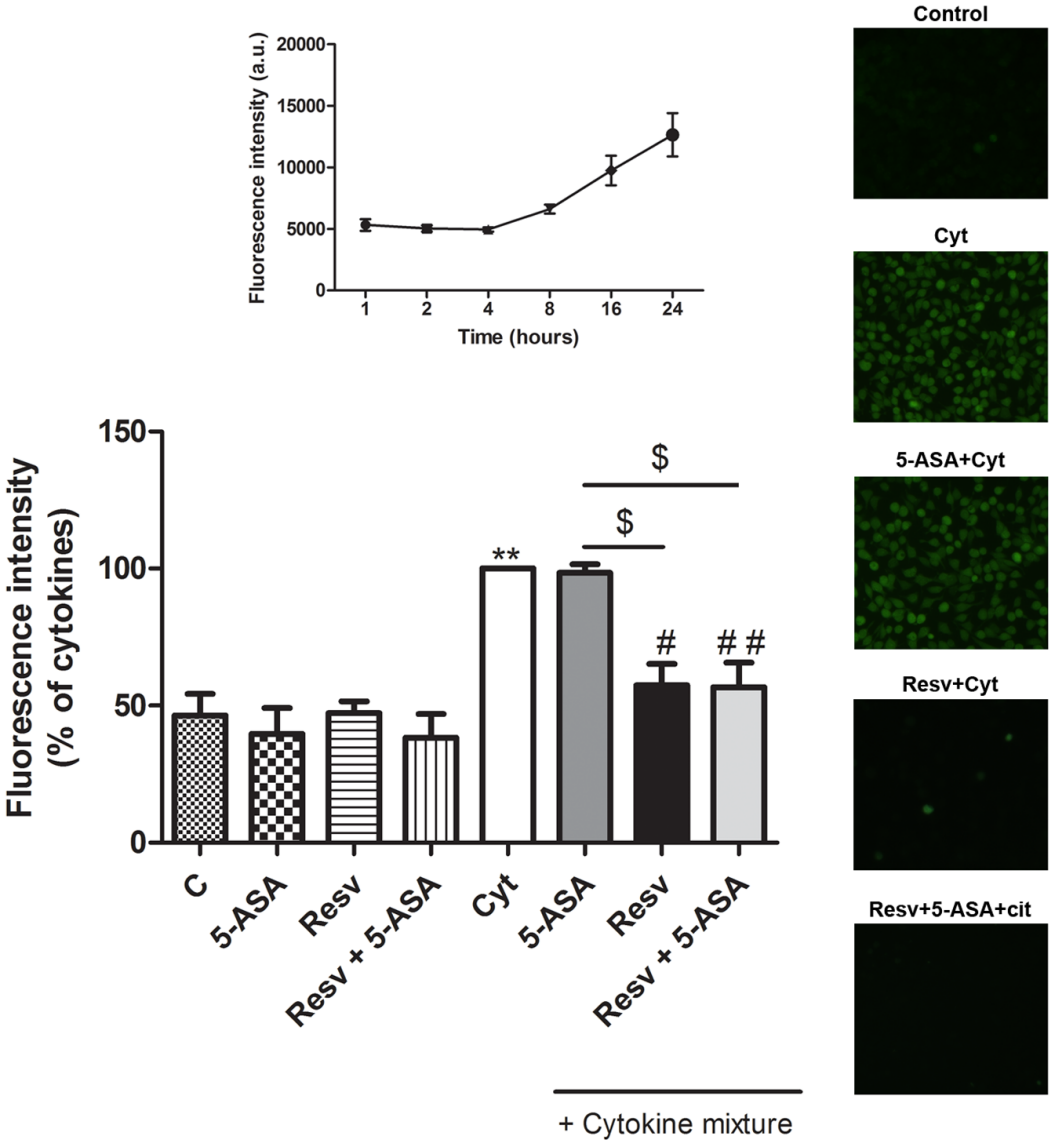
Resveratrol inhibits cytokine-induced oxidative stress in a greater extent than 5-ASA, in HT-29 cells. Cells were pre-incubated with 25 µM Resv or 500 µM 5-ASA or both (25 µM Resv plus 500 µM 5-ASA) for 1 hour and then exposed to a combination of cytokines for 16 hours. Reactive species production was measured after 16 hours of incubation with cytokines, by oxidation of the probe dichlorodihydrofluorescein, expressed in terms of fluorescence intensity relative to cytokine-stimulated HT-29 cells. A time-course of HT-29 reactive species production following cytokine challenge is presented on the top and representative images obtained by fluorescence microscopy (400×) of cells at 16 hours after cytokine treatment, in the absence or presence of 25 µM Resv and/or 500 µM 5-ASA are presented on the right. Values are mean ± SEM of at least three independent experiments, each one in duplicate. ^**^ *P*<0.01 vs Control, ^#^ *P*<0.05, ^##^ *P*<0.01 vs Cytokines and ^$^ *P*<0.05 vs ASA plus Cytokines.

It is well known that the formation of intracellular reactive species is close related to the inflammatory process. Thus, the ability of Resv, in comparison and associated with 5-ASA, to inhibit cytokine-induced intracellular oxidative stress was assessed by dichlorodihydrofluorescein fluorescence. A time-course analysis was carried out, following cytokine challenge. As shown on the top of Figure 9, the intracellular levels of reactive species started to increase after 8 hours of cell incubation with cytokines and were maintained until 24 hours. To assess the effects of Resv and/or 5-ASA, the experiment was conducted with 1 hour of cell pre-incubation with the compounds, followed by 16 hours of incubation with cytokines. As evidenced in the bar graph and typically shown in the pictures on the right of Figure 9, Resv exhibited a stronger efficiency as an antioxidant than 5-ASA, and the association Resv and 5-ASA seems not to potentiate the effect of Resv *per se*.

## Discussion

The growing knowledge on the cell signaling pathways, underlying the inflammatory process that characterizes Inflammatory Bowel Disease [22], [35], has allowed the investigation of better target strategies to limit IBD progression. However, despite the advances in the pharmacological treatment that have been made in the last years, this disease remains devoid of cure and, consequently, the pharmacological management is mainly used to prevent and to treat symptoms and still induce or maintain the remission periods [23]. On the other hand, given that IBD patients often become refractory to the existing therapies, several lines of evidence show that many patients take some form of dietary supplement to achieve extra benefits [23], [36].

The anti-inflammatory role of polyphenols in chronic inflammatory diseases as IBD has been supported by many authors, who believe that the consumption of these biological phytochemicals can be highly advantageous to prevent or limit disease progression [5], [6], [8]. Resveratrol is a non-flavonoid polyphenol, particularly abundant in grapes and red-wine, whose action mechanisms have been extensively studied in the last decades. However, one of the major concerns about Resv efficacy is related to its low oral bioavailability [16], [17]. In fact, it is estimated that after oral intake, Resv can be extensively metabolized in the liver and intestine, resulting in a very low bioavailability in humans [10], [16]. Nevertheless, there are evidences demonstrating that this compound is able to accumulate in specific tissues, particularly in the intestinal tissue, where its glucuronic acid and sulfate conjugates, the major metabolites of Resv, may work as a pool of the active compound that is released upon the action of β-glucuronidases or sulfatases [16], [37]. This suggests that Resv may be particularly attractive to modulate the inflammatory process settled in IBD patients.

In the present study, it was first examined the impact of Resv, alone or in combination with the pharmaceutical agent 5-ASA, on the HT-29 cell viability. Afterward, considering that beyond its bioavailability, another major concern about Resv helpfulness is related to some studies pointing out that the consumption of high doses of polyphenols may be deleterious to the liver [18], [38], the possible hepatotoxicity of our compounds was assessed on HepG2 cell line. To exclude any possible toxicity, the selected concentrations for the next experiments were 25 µM Resv and 500 µM 5-ASA. The reported capacity of Resv to reduce the levels of some pro-inflammatory mediators in macrophages [13] motivated us to verify its ability to counteract the induction of NO and PGE_2_ production by cytokines in HT-29 cells, comparing its action with that of the drug 5-ASA. Resv showed to be efficient in inhibiting cytokine-induced NO and PGE_2_ production, in a concentration 20 times lower than 5-ASA. Accordingly, in a study by Zhong *et al* [12], Resv showed similar effects in LPS-stimulated BV-2 microglial cells. The combination of Resv and 5-ASA did not give any evidence of enhanced efficiency, in our experimental conditions.

To assess whether the above protective effect of Resv, concerning pro-inflammatory mediators, was exerted via inhibition of iNOS and COX-2, both protein and mRNA production were evaluated. Our results showed that Resv can suppress transcriptionally the cytokine-induction of these two enzymes, in a greater extent than 5-ASA, revealing its anti-inflammatory superiority and, on the other hand, that the inhibition of iNOS and COX-2 are (at least in part) related to the reduction of NO and PGE_2_ production induced by Resv. This is consistent with the results previously reported by Cianciulli *et al* [39], showing the ability of Resv to inhibit LPS-induced COX-2 and PGE_2_ production, in Caco-2 cells.

The transcriptional regulation of pro-inflammatory markers is a strictly controlled event regulated by several transcriptional factors as NF-kB. NF-kB pathway is one of the most studied regulators of the transcription of pro-inflammatory genes, such as those of iNOS and COX-2. Besides, it is known that this pathway is usually induced in the intestine of IBD patients [29]. It turns out that in our experimental conditions, Resv could not prevent the degradation of IkB-α which is a well-established step for the classical activation of NF-kB pathway [29]. Our results are, hence, apparently in contradiction to those reported by Zhong *et al* [12], demonstrating the inhibition of LPS-induced activation of NF-kB pathway by Resv in microglial cells. In fact, in our study, Resv showed to be not able to down-regulate the activation of NF-kB pathway, either alone or in combination with 5-ASA.

Therefore, the anti-inflammatory effect of Resv should be explained by the involvement of an alternative cell signaling pathway, such as JAK-STAT pathway, which is also induced in IBD patients [32]. In a previous study from our group, it was demonstrated for the first time, the ability of 5-ASA to decrease the amount of activated STAT1 in the nucleus of HT-29 cells, by evaluating the levels of phosphorylated STAT1 at Tyr701 [8]. It is believed that after IFN-γ-receptor stimulation, STAT1 is phosphorylated at Tyr701, being this event essential for STAT1 dimerization, its translocation to the nucleus and DNA binding or, in other words, for STAT1 activation [40]. In the present study, Resv ability to reduce the levels of Tyr701 phosphorylated STAT1 in the nucleus of HT-29 cells was clearly demonstrated. The combination of Resv with 5-ASA showed a similar effect to that of the compounds alone. To our knowledge, no study dealing with the ability of Resv to reduce cytokine-increased levels of Tyr701 phosphorylated STAT1, in the nucleus of HT-29 cells, has been reported yet. Moreover, this finding is in line with the observation of Capiralla *et al* [41], demonstrating the suppressive effect of Resv on LPS-induced phosphorylation of STAT1 at Tyr701 in RAW 264.7 macrophages and BV-2 microglial cells.

The observed reduction of activated STAT1 accumulated in the nucleus induced by Resv in HT-29 cells, may have an extremely important impact in the context of IBD, since besides its implications for the inhibition of many pro-inflammatory genes, it can contribute for the prevention of radiation resistance acquired by IBD patients during radiotherapy treatment, which has been associated to high levels of nuclear STAT1 [42]. In fact, IBD patients exhibit a high risk of developing colorectal cancer as compared to healthy population [43] and, for this reason, the use of anti-inflammatory compounds with the potential to inhibit that event (acting as anticancer agents) can be of great importance in IBD context. Therefore, taking into account the range of concentrations at which Resv and 5-ASA exert anti-inflammatory effects, Resv proved to give further benefits in the context of IBD as compared to 5-ASA.

Seeking more information about the protective role of Resv as compared to 5-ASA, the involvement of MAPK pathway was studied. MAPKs are a group of enzymes considered as instigative controllers of many downstream signaling pathways, with relevance, for example, in the activation of some transcription factors [31]. The most known subfamilies of MAPKs are ERK 1/2, SAPK/JNK and p38 MAPK. ERK 1/2 is strongly activated by growth factors and in a lesser extent by cytokines [44]. In contrast, SAPK/JNK and p38 MAPK are strongly activated by cytokines, such as TNF-α [44]. This report shows that, contrarily to what happens with 5-ASA, Resv inhibited cytokine-induced phospho-SAPK/JNK levels, in HT-29 cells. The combination of Resv with 5-ASA seemed to enhance the ability of Resv to decrease the levels of this phosphorylated protein. Large body of evidence suggests that SAPK/JNK pathway is an important signal transduction pathway implicated in IBD [45] and for this reason there is a recent considerable interest in the development of anti-JNK therapies [45]. Towards this goal, several studies have been conducted demonstrating the effectiveness of some JNK inhibitors in the protection against pathophysiology features of experimentally induced IBD in some *in vivo* models [46], [47]. On the other hand, SAPK/JNK pathway is also involved in the phosphorylation of STAT1 at Serine 727 [48] and it is known that this specific phosphorylation is required for the maximization of the transcriptional potential of STAT1. In fact this post-translational modification is important, for instance, for the modulation of the interaction of STAT1 with co-activator proteins [49]. Thus, our present data suggest that the anti-inflammatory protection afforded by either Resv alone or in combination with 5-ASA involves the prevention of SAPK/JNK activation and the subsequent impairment of the maximization of STAT1 transcriptional potential. This is a meaningful finding in the light of the therapeutic potential of JNK inhibitors.

Besides, there is accumulating evidence that p38 MAPK can also mediate the STAT1 Ser727 phosphorylation [48], [50]. This event would enhance PIAS1 (protein inhibitor of activated STAT1) binding and SUMO-1 (small ubiquitin-related modifier-1) conjugation to STAT1 [51]. PIAS are a family of proteins implicated in the inhibition of STAT-mediated gene activation through many mechanisms, such as inhibiting DNA binding and promoting SUMO conjugation of STAT1 [40]. SUMOylation is a post-transcriptional modification that, in the case of STAT1, seems to function as a negative regulator, since inhibits STAT1 Tyr701 phosphorylation, prevents STAT1 DNA binding and also promotes its dephosphorylation [40], [51]–[53]. Thus, p38 MAPK induced maximization of STAT1 transcriptional potential (by phosphorylation on Ser727) and this event would precede the relatively slow emergence of STAT1 SUMOylation, triggering a negative feedback loop through PIAS1 and SUMO recruitment [52]. Remarkably, our data show that cytokine-induced activation (phosphorylation) of p38 MAPK was not counteracted by Resv and/or 5-ASA. This probably means that the protective effect of Resv or Resv plus 5-ASA takes advantage of the p38 MAPK-mediated negative feedback of STAT1. These findings strengthen our knowledge regarding the pathways involved in the protection afforded by Resv, 5-ASA and the combination of Resv with 5-ASA, in activated intestinal cells.

On the other hand, several studies demonstrate that oxidative stress and inflammation are closely related and therefore persistently elevated levels of ROS can contribute to the perpetuation of the inflammatory process and ultimately to cancer [54]. For this reason, we evaluated the effect of our compounds in the production of ROS. Interestingly, Resv pre-treatment of HT-29, before cytokine challenging, prevented the cytokine-induction of oxidative stress in these cells, which was not verified with 5-ASA pre-treatment. Thus, it is noteworthy that Resv has a more efficient antioxidant activity than 5-ASA in this type of cells.

In conclusion, under our experimental conditions, Resv revealed a stronger anti-inflammatory and antioxidant activity than 5-ASA, given that in a concentration 20 times lower, Resv was able to efficiently decrease cytokine-induced pro-inflammatory mediators (NO and PGE_2_) production, pro-inflammatory enzymes (iNOS and COX-2) expression and intracellular reactive species formation. Moreover, in spite of not being able to prevent cytokine-induced IkB-α degradation, Resv efficiently decreased the amount of Tyr701 phosphorylated STAT1 in the nucleus of HT-29 cells, suggesting that JAK-STAT pathway is one of the key cascades involved in its anti-inflammatory activity. On the other hand, in contrast to 5-ASA, Resv was also able to inhibit the activation of the SAPK/JNK pathway, preventing the transcriptional potential maximization of the remaining Tyr701-phosphorylated STAT1. Furthermore Resv takes advantage of the negative feedback of STAT1, thought p38 MAPK pathway.

Overall, Resv did not exhibit a synergistic effect with 5-ASA.

Put together, data collected from our previous work and gathered from the present work support our belief that two polyphenols with completely different chemical structures, Cyanidin-3-Glucoside and Resveratrol, abundant in a Mediterranean Diet, can assume a more efficient anti-inflammatory role than that of 5-ASA, a well-known pharmacological agent, used as the cornerstone of treatment for IBD patients. However, we are aware that the therapeutic value of these compounds must be confirmed by *in vivo* experiments, which are planned in a near future.

Considering that current treatment options for IBD patients are not completely successful, the Mediterranean Diet (rich in those polyphenolic compounds) can be envisaged as an interesting strategy to promote remission periods in IBD patients, limiting IBD progression and even to obviate colorectal cancer, which is commonly inflicted on these patients.
